# Cost-effectiveness of selective prophylactic mesh placement after stoma closure

**DOI:** 10.1007/s10029-025-03436-2

**Published:** 2025-08-27

**Authors:** Camilo Ramírez-Giraldo, Sofía Santamaría-Forero

**Affiliations:** 1 Hospital Universitario Mayor - Méderi, Calle 24 #29-45, Bogotá, Colombia; 2 Grupo de Investigación Clínica, Escuela de Medicina y Ciencias de la Salud, Universidad del Rosario, Bogotá, Colombia

**Keywords:** Stoma closure, Stoma reversal, Ileostomy reversal, Incisional hernia, Prophylactic mesh

## Abstract

**Background:**

Stoma closure is associated with a high incidence of incisional hernia, which not only impose significant costs on the healthcare system but also negatively impact patient’s overall health. Prophylactic mesh placement for stoma closure has proven effective in preventing these hernias; however, its broader implementation is limited primarily by cost-effectiveness concerns.

**Methods:**

We carried out an economic evaluation to assess the cost-effectiveness of the use of selective prophylactic mesh (high-risk patients) in patients who underwent stoma closure, using data from a systematic review and meta-analysis that evaluated risk factors, and another systematic review and meta-analysis that evaluated the effectiveness of prophylactic mesh.

**Results:**

Our study showed that selective strategy offers an alternative with a lower incremental cost and comparable, although lower effectiveness. While prophylactic mesh placement in all patients has a higher incremental cost, it also has higher effectiveness. Nevertheless, the incremental cost does not exceed the threshold established in our setting of 2 times the GDP per capita.

**Conclusion:**

The use of prophylactic mesh for the closure of all stomas is a strategy that should be implemented and performed with a prosthetic mesh to be a cost-effective strategy.

## Introduction

Following stoma closure, incisional hernias develop at the former stoma site in approximately 20–40% of cases [1]. These hernias significantly reduce quality of life, impair physical function, and increase both morbidity and mortality, while also imposing a substantial economic burden on healthcare systems [2, 3]. To address this, the use of prophylactic mesh placement has been evaluated, with studies showing a reduction in the incidence of incisional hernias without a corresponding increase in postoperative complications [4].

However, based on the cost-effectiveness findings from the ROCSS randomized controlled trial, prophylactic mesh placement after stoma closure does not appear to be a cost-effective preventive strategy in the general population [5]. In contrast, other cost-effectiveness studies assessing prophylactic mesh use during laparotomy in high-risk patients have demonstrated favorable economic and clinical outcomes [6]. These findings suggest that prophylactic mesh may become cost-effective in the context of stoma closure if used selectively in high-risk patients. Such patients include those with known risk factors for incisional hernia development, such as elevated body mass index (BMI), preexisting parastomal hernias, and/or terminal colostomies [7]. An additional factor influencing cost-effectiveness is the type of mesh used. In the ROCSS trial, a biological mesh was employed, which is substantially more expensive than synthetic alternatives [8].

In light of the above, the objective of this study was to evaluate the cost-effectiveness of selective prophylactic placement of synthetic mesh in patients undergoing stoma closure.

## Materials and methods

### Study design

An economic evaluation was conducted to assess the cost-effectiveness of synthetic prophylactic mesh placement in patients undergoing stoma closure. The analysis compared mesh placement versus no mesh, either in high-risk patients (selective use) or in all patients, taking into account procedure-related costs and hernia recurrence during follow-up. Risk factors and the probability of hernia recurrence were identified and modeled using data from two systematic reviews: one systematic review and meta-analysis evaluating risk factors for incisional hernia after stoma closure, and another assessing the effectiveness of prophylactic mesh placement. This study was reported in accordance with the CHEERS (Consolidated Health Economic Evaluation Reporting Standards) guidelines [9].

The systematic review and meta-analysis that evaluated risk factors included 2455 patients, found a 16.76% incidence of incisional hernia after stoma closure, and identified that an elevated body mass index (OR = 1.95, 95%CI = 1.41–2. 69), presence of parastomal hernia (OR = 3.42, 95%CI = 1.55–7.61), colostomy (OR = 2.03, 95%CI = 1.52–2.74) and terminal stoma (OR = 1.46, 95%CI = 1.02–2.08) were risk factors for development of incisional hernia at the stoma site after stoma closure [7].

The systematic review and meta-analysis evaluating the effectiveness of prophylactic mesh included 2008 patients, where an incidence of incisional hernia was found to be 12.38% after stoma closure. Prophylactic mesh placement was a protective factor for the development of incisional hernia (OR = 0.21, 95%CI = 0.12–0.37). There were no statistically significant differences in terms of operative site infection, hematoma, seroma or reinterventions between those who underwent mesh and those who did not [10].

Costs were measured in monetary units (Colombian pesos. 1 USD = ~ 4025 Colombian pesos), and effectiveness was expressed in terms of incisional hernias avoided. The analysis was conducted from the perspective of the Colombian healthcare system, excluding the patient perspective. The time horizon was determined based on the follow-up periods reported in the included meta-analyses: for the meta-analysis evaluating risk factors, follow-up ranged from 4.4 to 68.4 months; for the meta-analysis assessing the effectiveness of prophylactic mesh, the range was 10 to 33 months. All costs were calculated in 2025 Colombian pesos and, therefore, no discounting was applied.

### Description of the model

The main outcome of the study was the absence of incisional hernia. A decision tree was performed comparing the incidence of incisional hernia in low-risk and high-risk patients, and in high-risk patients who did and did not undergo prophylactic mesh placement. The parameters were based on the probability of developing incisional hernia after stoma closure estimated from two systematic reviews and meta-analyses that evaluated risk factors and the effectiveness of prophylactic mesh.

To define the arm at low or high risk of developing an incisional hernia according to the risk factors, the incidence of incisional hernia and the risk factors identified as statistically significant in the systematic review and meta-analysis were taken into account [7], and an estimate of the baseline risk was made from the following model:

$$\:logit\left(P\right)=\beta0+\beta1\left(X1\right)+\beta2\left(X2\right)+\beta3\left(X3\right)+\dots\:\beta k\left(Xk\right)$$

Where,

$$\begin{array}{l}logit\left(Incisional\:hernia\:incidence\right)=\beta0+\beta1\left(BMI\right)+\beta2\left(colostomy\right)\\+\beta3\left(terminal\:ostomy\right)+\beta4\left(parastomal\:hernia\right)\end{array}$$

We clear,

$$\begin{array}{c}\beta0=\mathrm{log}\left(\frac P{1-P}\right)-(\beta1\left(BMI\right)+\beta2\left(colostomy\right)+\\+\beta3(terminal\:ostomy)+\beta4\left(parastomal\:hernia\right)\end{array}$$

Taking into account the incidence, the coefficients (beta), the BMI mean and the present proportion of risk factors (values obtained from meta-analyzing the means and proportions of the included studies using a random-effects model) we obtain:

$$\:\beta0=\mathrm{log}\left(\frac{0.1676}{1-0.1676}\right)-(0.66\left(26.41\right)+0.70\left(0.53\right)+0.37(0.51)+1.22\left(0.11\right))$$

$$\beta0=-19.94$$

The model calculates the probability of developing incisional hernia after stoma closure (the values for terminal stoma, colostomy and parastomal hernia are 1 when present and 0 when absent; we took a reference BMI value of 26.41 kg/m^2^):

$$\begin{array}{l}Predicted\:probability=\\\frac1{1+exp^{-(-19.94+0.66\left(BMI\right)+0.70\left(colostomy\right)+0.37(terminal\:stoma)+1.22\left(parastomal\:hernia\right)}}\end{array}$$

**Fig. 1:**
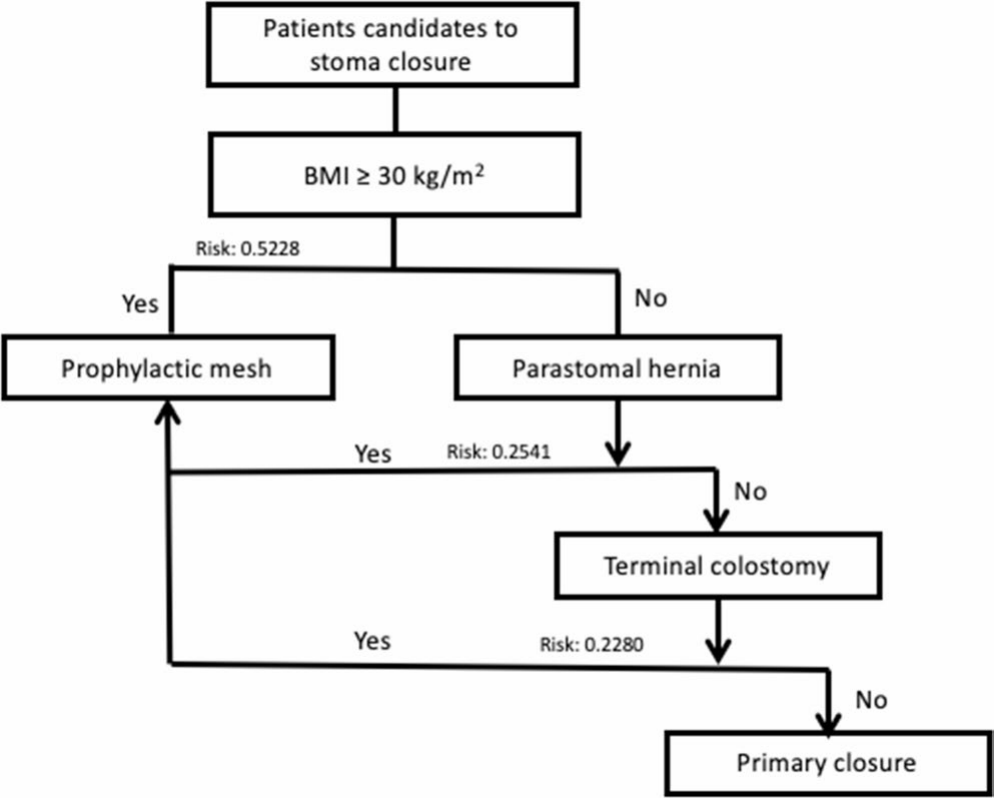
Algorithm proposal for selective mesh placement

Based on this we get a baseline risk of 0.0906. In case of a BMI of 30 kg/m^2^ in the absence of other factors the risk is 0.5228, if there is a parastomal hernia in the absence of other factors the risk is 0.2541, and if it there is a terminal colostomy in the absence of other factors the risk is 0.2280; if there are more of these factors in the same patient, the risk would be even higher. A proposed algorithm was designed (Fig. 1) to select the patients who would benefit from prophylactic mesh after stoma closure. To perform the cost-effectiveness analysis, the lowest probability of presenting incisional hernia was taken, that is, 0.2280.

The next arm was the use or non-use of prophylactic mesh in high-risk patients; the intervention was considered successful if the patient did not develop incisional hernia. This was determined from a systematic review and meta-analysis that evaluated the effectiveness of prophylactic mesh in preventing incisional hernias [10]. Similarly, the baseline risk (absence of prophylactic mesh) was calculated from the following model:

$$\:logit\left(P\right)=\beta0+\beta1\left(X1\right)$$

Where,

$$\:logit\left(P\right)=\beta0+\beta1\left(Prophylactic\:mesh\right)$$

We clear,

$$\beta0=\mathrm{log}\left(\frac P{1-P}\right)-\left(\beta1\left(Prophylactic\:mesh\right)\right)$$

Taking into account the incidence of incisional hernia in the studies in which mesh usage was evaluated, the coefficient (beta) of mesh use, and the proportion of mesh use we obtain:

$$\begin{array}{c}\beta0=\mathrm{log}\left(\frac{0.1238}{1-0.1238}\right)-(-1.56)\left(0.34\right))\\\beta0=-1.42\end{array}$$

The model calculates the probability of developing incisional hernia after the stoma closure with or without mesh placement (the value when mesh is absent is 0 and 1 when mesh is present):

$$Predicted\;probability=\frac1{1+exp^{-(-1.42+(-1.56\left(prophylactic\:mesh\right))}}$$

This gives a baseline risk of 0.1939 without the use of prophylactic mesh. If prophylactic mesh is used, the risk is 0.0481.

**Fig. 2:**
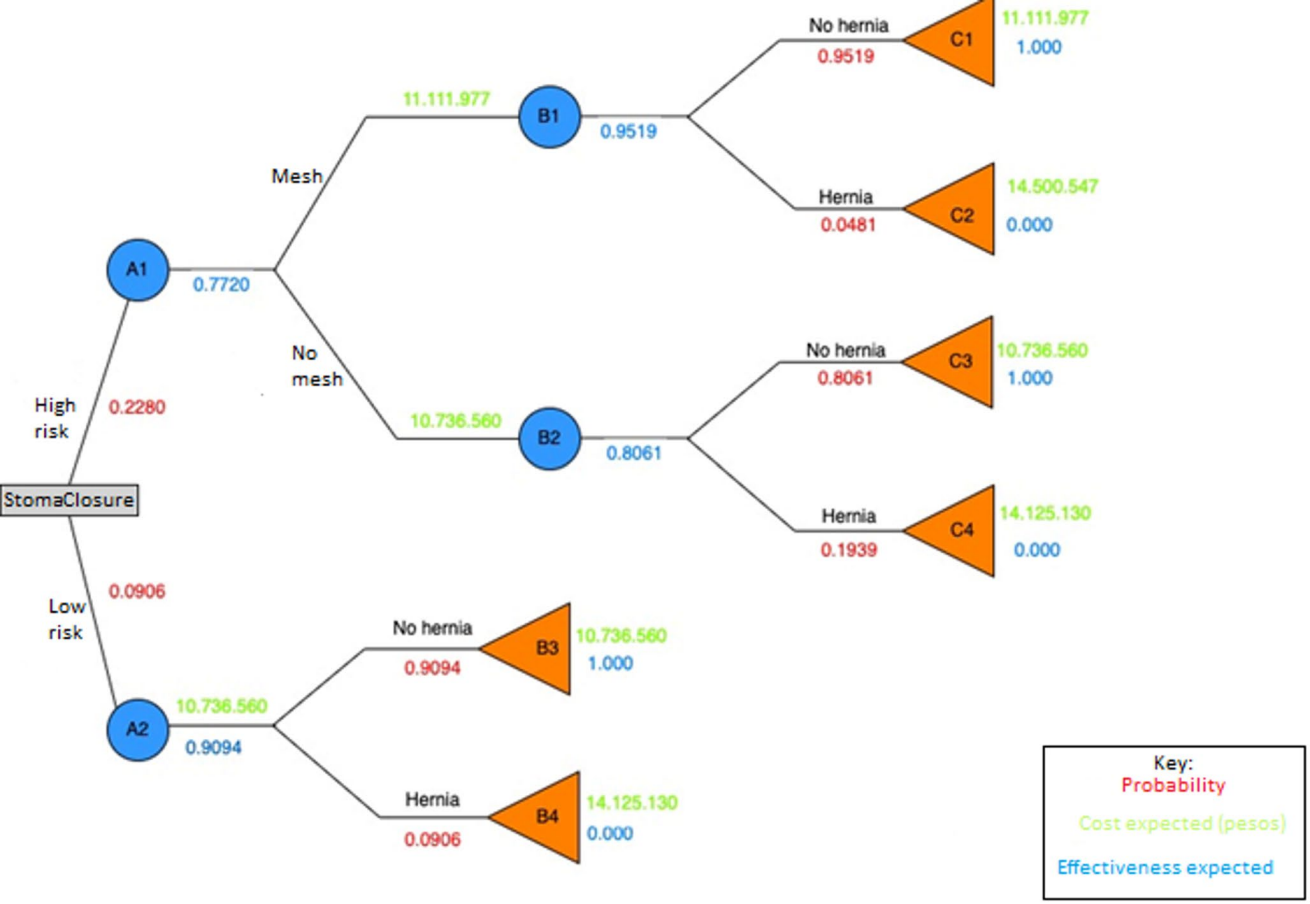
Decision tree showing probabilities, costs and effectiveness of events

Using the previously obtained probabilities, we can construct the following decision tree (Fig. 2), complications were excluded from consideration, as there was no statistically significant difference between the prophylactic mesh placement group and the control group.

Based on the developed models, a Monte Carlo simulation was performed based on individual patients, which enabled the cost-effectiveness evaluation of each strategy for preventing incisional hernias. Finally, we carried out a probabilistic sensitivity analysis using Monte Carlo simulation.

### Costs

The costs used in this analysis were estimated from a private hospital and included: the approximate cost of surgical closure of a stoma, the cost of hernia repair with prosthetic mesh, the cost of stoma closure with simultaneous placement of prosthetic mesh, as well as the costs associated with consultations with both the anesthesiology service and general surgery.

### Effectiveness

Effectiveness was presented as the number of incisional hernias prevented per stoma closure performed. Probabilities were obtained from the models previously described.

## Results

### Cost-effectiveness

We carried out a Monte Carlo simulation with 100.000 simulations, assuming 50% high-risk patients, and the already known risks for developing hernia according to whether it is a high-risk or low-risk group and whether or not prophylactic mesh was used, and also with the costs described.

The average cost in case of performing the strategy of treating all patients with prophylactic mesh would be 11.273.747 Colombian pesos with an effectiveness of 0.9522, the strategy of only treating high-risk patients with prophylactic mesh would be of 11.163.614 Colombian pesos with an effectiveness of 0.9290 and the strategy of not placing prophylactic mesh would be 10.736.560 Colombian pesos with an effectiveness of 0.8061 (Table 1).

**Table 1:** Cost-effectiveness comparison of the different strategies to prevent incisional hernias after stoma closure

Prophylactic mesh for everyone versus Selective prophylactic mesh		
ΔCost	= 11.273.747 − 11.163.614	= 110.133 pesos
ΔEffectiveness	= 0.9522 − 0.9290	= 0.0232
ICER	= 110.133/0.0232	= 4.747.112 pesos for prevented hernia
Prophylactic mesh for everyone versus No mesh		
ΔCost	= 11.273.747–10.736.560	= 537.187 pesos
ΔEffectiveness	= 0.9522–0.8061	= 0.1461
ICER	= 537.187/0.1461	= 3.679.323 pesos for prevented hernia
Selective prophylactic mesh versus No mesh		
ΔCost	= 11.163.614–10.736.560	= 427.054 pesos
ΔEffectiveness	= 0.9290–0.8061	= 0.1229
ICER	= 427.054/0.1229	= 3.475.945 pesos for prevented hernia

**Fig. 3:**
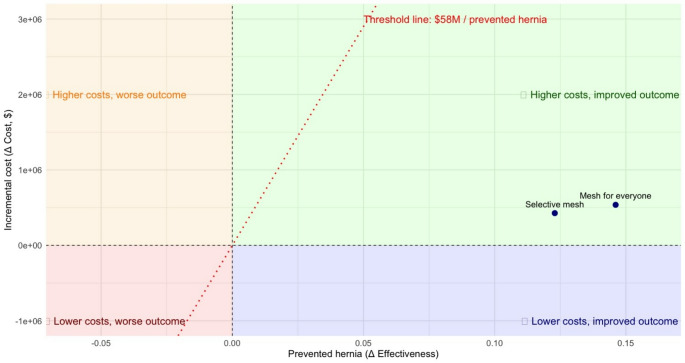
Cost-effectiveness plane of prophylactic mesh use for everyone versus selective prophylactic mesh

Both strategies, placement in all patients and selective placement of prophylactic mesh after stoma closure were cost-effective, according to the threshold of 2 times the GDP per capita of Colombia (~ 58.000.000 pesos). The ICER for these two strategies is projected on a cost-effectiveness plane (Fig. 3).

### Sensitivity analysis

To evaluate the robustness of the model, a Monte Carlo simulation was performed with 10,000 iterations, incorporating variability in the hernia probabilities and in the costs of the strategies. In each iteration, the costs and effectiveness of the two strategies compared were calculated: prophylactic mesh placement in all patients and selective placement only in high-risk patients.

**Fig. 4:**
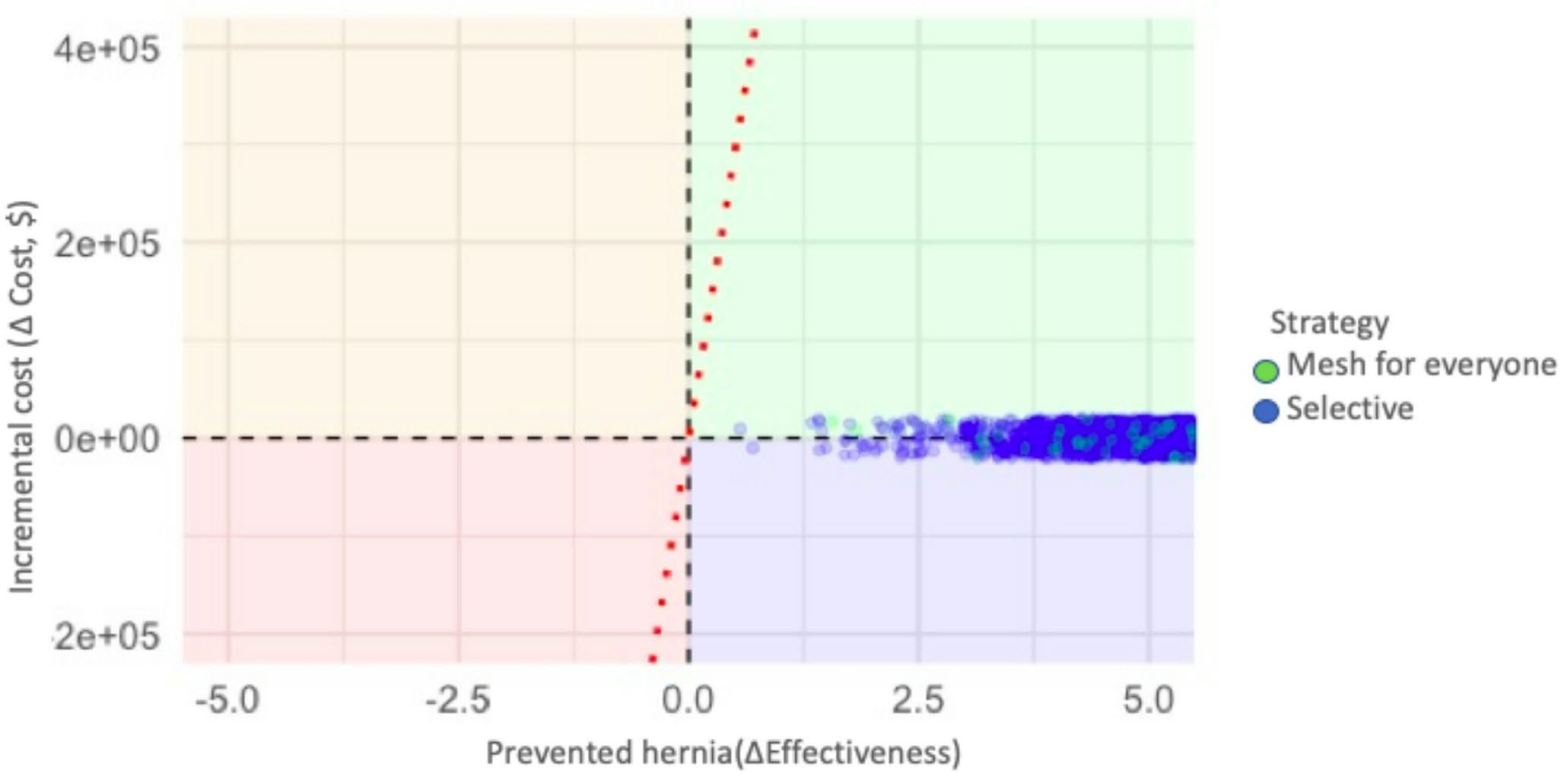
Sensitivity analysis with Monte Carlo simulation prophylactic mesh for everyone vs. selective prophylactic mesh

The results were visualized in a cost-effectiveness plane (Fig. 4), where each point represents an individual simulation. Both groups were located between the upper right and lower right quadrant, indicating that both strategies generate a benefit in effectiveness, at a similar or slightly higher cost.

The points for both strategies were below the cost-effectiveness threshold of 58 million pesos per hernia avoided, suggesting that both options would be cost-effective in our context.

## Discussion

Our study showed that selective strategy offers an alternative with a lower incremental cost and comparable, although lower, effectiveness. Taking these findings into account, we consider that the best strategy in our setting would be prophylactic mesh placement in all patients. This is because, although it has a higher incremental cost, it also has higher effectiveness. This higher cost does not exceed the threshold established in our setting of 2 times the GDP per capita, and furthermore, they are still similar. Therefore, we believe that it is worth this small additional cost at the expense of slightly higher effectiveness.

Prophylactic mesh placement in patients undergoing stoma closure has been widely debated, not only for its potential benefits and risks but also for concerns about cost-effectiveness and added operative time. However, some studies have found no significant difference in operative time between the two approaches [11].

Previously, a cost-effectiveness study had been performed based on the randomized clinical trial ROCCS that compared the placement of prophylactic biological mesh versus non-placement, in which a lower risk of incisional hernia was evidenced with the placement of prophylactic biological mesh [12]. Despite the effectiveness of the therapy in the clinical trial, the cost-effectiveness study failed to show that it was cost-effective [5]. This could be due to many factors, but one of them was the high cost of the meshes. Biological meshes have a cost, which has been determined to be up to 200 times higher than prosthetic mesh [8].

In addition to ROCSS, there are studies that directly compare the use of prophylactic biological mesh and prophylactic synthetic mesh in loop ileostomy reversal, evaluating parameters such as incisional hernia development and other complications associated with the use of mesh such as seroma, infection, recurrence and others. These have shown that the use of synthetic meshes has been much more cost-effective than biological meshes, in addition to the fact that they are highly effective in preventing incisional hernias and that the complications associated with the use of this type of mesh are not high in comparison with biological meshes [13, 14].

It is necessary to perform a cost-effectiveness and cost-utility study in patients who underwent stoma closure with prosthetic mesh as prophylaxis versus no mesh placement. Additionally, the readmission of patients for different complications associated with both ostomy reversal and the use of prophylactic mesh should be considered to estimate the total cost.

This study has several limitations. First, a cost-utility analysis was not performed because the outcomes reported in the systematic reviews used for the simulation were not expressed in quality-adjusted life-years. Second, the economic evaluation did not incorporate the patient perspective. Third, the analysis was based on simulations derived from previous systematic reviews, with cost estimates specific to the Colombian healthcare system, which may limit the generalizability of the results to other settings. Furthermore, our evaluation only considered hernias occurring at the stoma closure site, without taking into account the possible development of hernias at other incision sites associated with laparotomy or laparoscopy. Such additional hernias could lead to further costs and impact the overall economic results, representing another limitation to the comprehensiveness of our analysis.

## Conclusion

The use of prophylactic mesh during the closure of all stomas is a recommended strategy and should be carried out using prosthetic mesh to ensure cost-effectiveness. When it is not feasible to apply this approach to all patients—due to logistical, economic, or material constraints—should be prioritized for high-risk patients, where it remains a cost-effective strategy.
